# P-1309. Comparative Treatment Outcomes of Non-Extended-Spectrum Beta-Lactamase (ESBL), Ceftriaxone Non-Susceptible, Enterobacterales Bacteremia with Piperacillin/Tazobactam, Cefepime, Carbapenem or Other Antibiotic Treatment

**DOI:** 10.1093/ofid/ofaf695.1497

**Published:** 2026-01-11

**Authors:** Esther Kanner, Sumeet Jain, Pranisha Gautam-Goyal, Aya Haghamad, Tungming Leung, Vincent Streva, Jamie Lemon, Patricia Saunders-Hao

**Affiliations:** Northwell Health, Brooklyn, NY; North Shore University Hospital, Westbury, NY; Zucker School of Medicine at Hofstra/Northwell, Manhasset, New York; Northwell, Lake Success, New York; Northwell Health, Brooklyn, NY; Northwell Health Laboratories, Queens, NY; Northwell Health Laboratories, Queens, NY; North Shore University Hospital, Westbury, NY

## Abstract

**Background:**

Treatment of ceftriaxone-resistant Enterobacterales is largely focused on ESBLs, with a carbapenem being the preferred treatment. A subset of ceftriaxone resistant Enterobacterales are non-ESBLs. These organisms test negative for both genotypic and phenotypic detection of ESBLs. There is a paucity of data surrounding optimal management of this unique infection.

**Figure:**
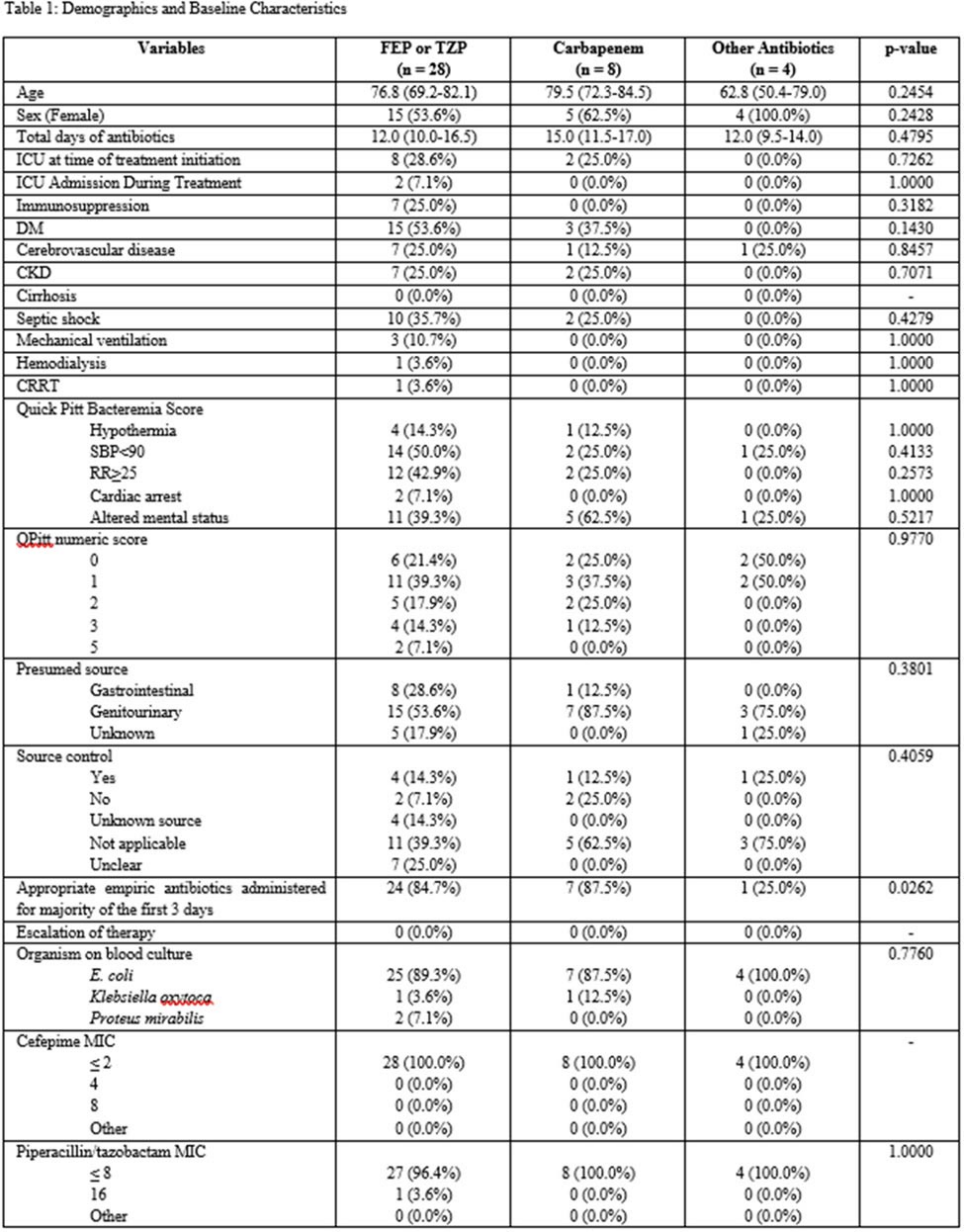

**Figure:**
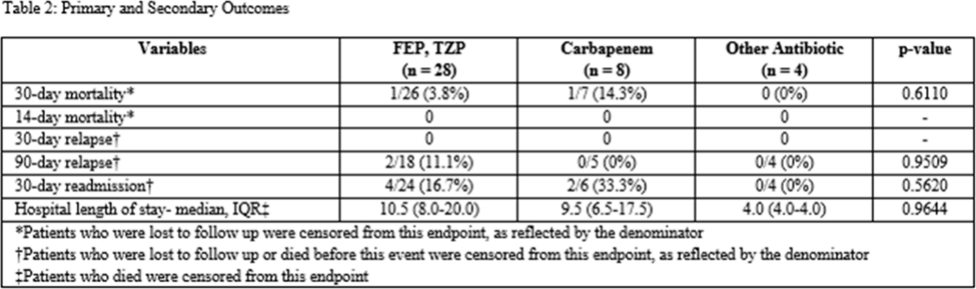

**Methods:**

This retrospective observational chart review evaluated adult patients admitted between January 2016 and November 2024 with a bacteremia caused by a non-ESBL, ceftriaxone-resistant, piperacillin/tazobactam (TZP)-sensitive, cefepime (FEP)-sensitive *Escherichia coli, Klebsiella oxytoca, Proteus mirabilis*, or *Klebsiella pneumoniae*. Exclusion criteria included bacteremias caused by the same organism within 6 months prior to the index blood culture, polymicrobial bacteremia, treatment without intent to cure, carbapenem resistance, antibiotics with Gram-negative bacterial coverage for another indication, pregnancy or lactation, or patients who expired within 48 hours of positive cultures. Patients were stratified into 3 groups depending on the administered targeted treatment (FEP or TZP vs. carbapenem vs. other).

**Results:**

Of the 76 patients who were screened, 44 met the inclusion criteria. Four patients received a resistant cephalosporin for targeted treatment and were excluded from the analysis. There were 28 patients in the FEP or TZP group, 8 patients in the carbapenem group, and 4 patients in the other antibiotics group. The median age of the cohort was 77 years, most patients were female, and 30% of patients were in septic shock. The most common source of infection was genitourinary (63%). Thirty-day mortality was 3.8% in the FEP or TZP group, 14.3% in the carbapenem group, and 0% in the other antibiotic group (p=0.611). There was no significant difference in any of the secondary outcomes.

**Conclusion:**

In this retrospective chart review study, there was no difference between carbapenem and non-carbapenem treatment for ceftriaxone-resistant, non-ESBL Enterobacterales bacteremia. Collaboration with other healthcare systems to increase sample size may help assess optimal treatment of these organisms.

**Disclosures:**

All Authors: No reported disclosures

